# AMAP assessment 2015: human health in the Arctic

**DOI:** 10.3402/ijch.v75.33949

**Published:** 2016-12-13

**Authors:** Jon Øyvind Odland, Shawn Donaldson, Alexey Dudarev, Anders Carlsen

**Affiliations:** 1 Department of Community Medicine, Faculty of Health Sciences, UiT The Arctic University of Norway, Tromsø, Norway; 2 Health Canada, Ottawa, ON, Canada; 3 Northwest Public Health Research Center, St. Petersburg, Russia; 4 Den Danske Miljøstyrelsen, Copenhagen, Denmark

The 2015 Human Health Assessment Report has continued the historical line from the former reports (1–3), including new knowledge, missing links and information from each report, drawing attention to the most recent knowledge and perspectives for future research. The separate chapters in this special issue reveal details on monitoring, known health effects, relations to climate change and, not least, the difficult communication challenges to reach policymakers, public health authorities and the people of the Arctic.

The Arctic Monitoring and Assessment Programme (AMAP), Phase 1, started in 1991 to implement components of the Arctic Environmental Protection Strategy (AEPS) as adopted at that time by Ministers of the eight Arctic countries. The main task was to prepare an assessment of the state of the Arctic environment with respect to defined pollution issues. On the basis of this, AMAP designed and implemented a monitoring programme, and conducted its first assessment. The monitoring programme was largely based on adaptation of ongoing national and international activities, initiating new monitoring and research work only where necessary. The first AMAP assessment was presented in 1998, entitled “Arctic Pollution Issues: A State of the Arctic Environment Report” (1). The first AMAP assessment was a compilation of current knowledge about the Arctic region and a statement of the prevailing conditions in the area. The report had a broad and holistic perspective, with human health as a separate chapter. The report would have been impossible without the generous offer from Health Canada to analyse blood samples from all eight Arctic countries, providing the very first quality assured comparison of persistent organic pollutants (POPs) and metals in human biological materials at the circumpolar level. This basic biomonitoring programme was thoroughly discussed by Odland and Nieboer (4). Even though programmes implemented in scarcely populated areas provide special challenges, different programmes have been introduced in several regions and countries. It was concluded in the health chapter of the 1998 report that several groups of people in the Arctic are highly exposed to environmental contaminants. It was also concluded that variation in human exposure depends on a combination of (a) varying environmental concentrations of contaminants, (b) local physical and biological pathways which make the contaminants available and (c) the local dietary habits of the people. The overall report concluded that the current understanding of transport processes and the ability to quantify them was inadequate. In particular, the determination of transport processes and their relative importance or magnitude within and between compartments (air, land, water, ice, sediments and biota) is essential. One important recommendation was the well-known benefits of breast milk and traditional food against the suspected but not yet fully understood effects of contaminants. The importance of good and reliable biomonitoring programmes was also addressed. A special part of the 1998 report was the description of health status and living conditions of population groups living in the Arctic. This overview has been developed into a comprehensive set of circumpolar health indicators published by Young and Bjerregaard (5).

The 2002 report (2) went deeper into health effects, introducing case studies in different geographical areas. It focused upon the combined effects of “multiple environmental stressors”. Evidence from analyses of banked blood samples from Norway (non-Arctic donors) demonstrated an exponential increase in polybrominated diphenyl ethers since 1977. Progress was made in studies on the interactive effects of current levels of mixtures of POPs in the traditional diet. Also, information on concentrations found in various organs of species used for food had improved. These findings improved the basis for dietary advice aiming at reducing exposure (2).

The success of carefully developed public health strategies was demonstrated in the Faroe Islands where interventions related to consumption of pilot whale meat resulted in an 80% reduction in mean mercury body burdens. New methodologies were found for integration of epidemiological and mechanistic biomarker effect studies on human samples making it possible to estimate the effects of current exposure levels, the possible interactions and the modifying effects of nutrients (combined effects).

The 2009 report demonstrated an important advance in our understanding (3). Several of the ongoing mother/child cohorts provided results of effect studies, the epidemiologic design was improved and the basic molecular research was in rapid development. Risk communication and risk management were on the public health agenda. Human adaptation to climate change became part of the discussion, and several new substances emerged as contaminants of potential concern. Especially, recent data for Arctic Russia were described, indicating elevated levels of oxychlordane and polychlorinated biphenyls in indigenous coastal peoples from Chukotka. These levels were linked to consumption of marine mammals by these coastal peoples. Dichlorodiphenyldichloroethylene, the major metabolite of the pesticide dichlorodiphenyltrichloroethane (DDT), was now found at the highest concentration in Arctic Russia. This indicates that the likely source is recent use of DDT in Russian agriculture or as a pesticide in northern communities. Mercury levels were declining in many populations across the Arctic; however, Inuit people still had blood mercury levels 3 to 10 times higher than populations who consume imported foods. This finding was especially prominent in Greenland and parts of Arctic Canada. A decreasing proportion of Inuit women of childbearing age exceeded guidelines for blood mercury. Dietary changes due to social, cultural and economic changes, as well as people's responses to risk management recommendations in the Arctic, are likely to be the reasons for these decreases in human body burdens. A number of health effects, at molecular level, as well as in clinical studies, are now described and presented to public health authorities. This leaves a big challenge for the policymakers and public health workers. Risk communication must be carried out with great care and must be sensitive to cultural preferences at a community level. The AMAP Human Health Assessment Group has clearly stated that it cannot provide specific public health advice in local and regional situations, but can evaluate the circumpolar impacts of efforts by local health authorities to develop and disseminate advice.

This special issue will give you a thorough update of all aspects of human health in the Arctic, with focus on contaminant levels, health effects, future needs and how we can communicate our difficult scientific language to common people and health authorities. The authors are all members of the AMAP, Human Health Assessment Group. There are still many “black holes” in our assessment, and our ongoing and planned cohorts are open for new possibilities in Arctic health studies and public health advice. The basic aim is to provide knowledge for health promotion for all Arctic people.

The papers have been thoroughly peer reviewed following the guidelines of the journal and the AMAP. We are also happy to include a short paper on reproductive health in Yakutia, in addition to the papers based on the AMAP report. All papers stand on their own feet and can be read separately. We wish you a very interesting reading.

*Jon Øyvind Odland* Department of Community Medicine Faculty of Health Sciences UiT The Arctic University of Norway Tromsø, Norway Email: jon.oyvind.odland@uit.no *Shawn Donaldson* Health Canada, Ottawa ON, Canada *Alexey Dudarev* Northwest Public Health Research Center St. Petersburg, Russia *Anders Carlsen* Den Danske Miljøstyrelsen Copenhagen, Denmark
